# The elusive meningococcal meningitis serogroup: a systematic review of serogroup B epidemiology

**DOI:** 10.1186/1471-2334-10-175

**Published:** 2010-06-17

**Authors:** Vanessa N Racloz, Silva JD Luiz

**Affiliations:** 1 Swiss Tropical Institute, Socinstrasse 57, 4002, Basel, Switzerland; 2 Novartis Vaccines, 350 Massachusetts Avenue, Cambridge, MA 02139, USA

## Abstract

**Background:**

Invasive meningococcal disease (IMD), is a widely distributed, complex human disease affecting all age categories. The causative agent, *Neisseria meningitidis*, is spread through aerosol respiratory droplets. 13 different serogroups have been identified, each with varying epidemiological features including prevalence, virulence, immunogenicity, geographical and temporal distribution. Although preventative measures are available for several of the serogroups, meningococcal disease caused by serogroup B is of particular interest due to the challenge it presents concerning vaccine development.

**Methods:**

A systematic review of peer reviewed studies and reports, the collection of data from national and international health resources, along with the analysis of the Multi Locus Sequence Typing database was carried out aimed at collecting information concerning serogroup B IMD and the epidemiology attached to it.

**Results:**

A continuous output of related and novel STs occurring worldwide in terms of the hypervirulent clonal complexes was observed both in published studies and the MLST database in this case using the eburst software, which highlights the genetically diverse nature of serogroup B strains.

**Conclusions:**

With the recent dominance of serogroup B IMD seen in many countries, along with the presence of antibiotic resistance, vaccine development needs to target areas of the bacterium which tackle this widespread and heterogeneous aspect of meningococcal meningitis disease.

## Background

Invasive meningococcal disease (IMD) is a widely distributed, complex human disease affecting all age categories. As a naso-pharynx commensal bacterium, *Neisseria meningitidis *is spread through aerosol respiratory droplets and under circumstances yet unclear, can progress from a carriage state to IMD.

IMD and the economic burden associated with it is of significant importance for public health, not only in the epidemic prone regions, but also in areas with sporadic and hyperendemic forms of the disease [1,2].

**Figure 1:**
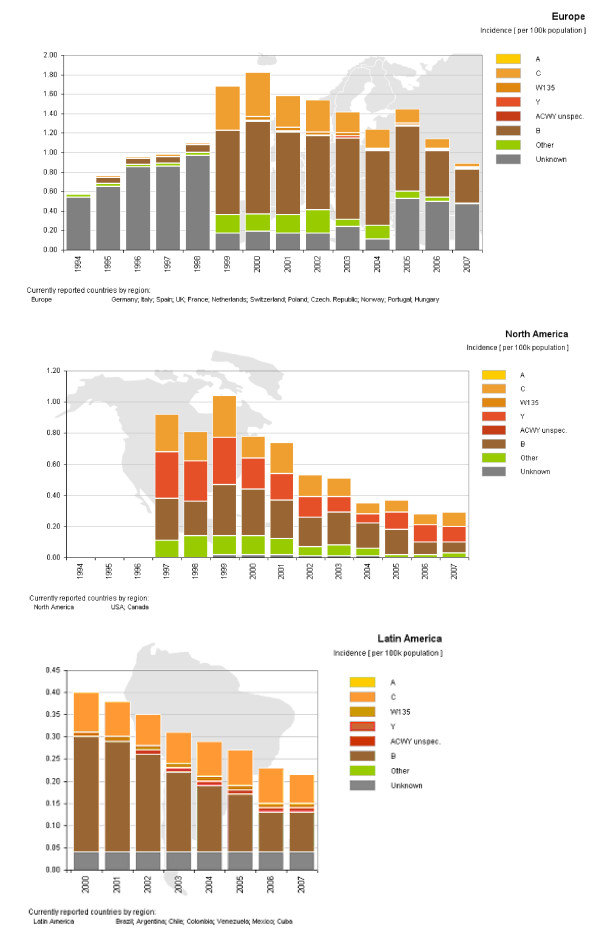
**Incidence of Meningococcal Disease by serogroup**. Distribution of meningococcal meningitis serogroups in the different regions present throughout 1994-2007. Colours correspond to the mentioned serogroups. Incidence given per 100,000 population.

According to differences in the chemical composition of the bacterial polysaccharide capsule, 13 different serogroups have been identified, each with varying epidemiological features including prevalence, virulence, immunogenicity, geographical and temporal distribution. Meningococcal disease caused by serogroup B is of particular interest due to the challenge it presents concerning vaccine development. Historically, serogroup A, and to a lesser extent C, have been the main causes of large epidemics as well as pandemics, mostly in Africa, Asia and Southern America. Nevertheless, serogroup B has posed a more recent threat with sporadic, endemic and epidemic occurrences being recorded in North America, Europe, South America and Australasia [3,4]. Since the introduction of vaccines against serogroups A and C, serogroup B has emerged as an important cause of IMD in regions such as Europe, Latin America and Northern America as seen in figure 1, especially due to the lack of preventative measures for this serogroup. More specifically, reports of major serogroup B epidemics started to surface during the second half of the 20^th ^century in Iceland and Norway (1976-86) [5], Turkey [6] as well as Cuba (1976) [7]. These epidemics were followed by outbreaks in Chile 1983-1987 [8], Brazil [9], the Netherlands [10], Belgium [11] along with New Zealand in the 1990s [12].

Since the 1990s, localized outbreaks have been reported in Oregon, USA where a hyperendemic situation was reported between 1993-1997, and has persisted albeit with a decreased incidence until the present day [13]. In the Seine-Maritime department, France [14] a hyperendemic situation has also been reported since 2003 attributed to the serogroup B ST32 (ET 5) complex also dominating in Oregon. In addition to the increasing importance of serogroup B in Europe and Northern America which has been rising steadily in the past decade [3,4], recent studies in Asia have also reported this serogroup as the dominant one as seen in Taiwan and Japan [15,16]. For the analysis of serogroup B data, understanding the currently used molecular typing methods is essential. Data obtained from the MLST database as described in the MLST typing home page have been used in the present study http://www.mlst.net[17].

Evolutionary developments can be analysed using this method in combination with the e-BURST (Based Upon Related Sequence Types) application as described below. MLST is based on strain characterization by sequencing internal fragments of seven housekeeping genes: abcZ (putative ABC transporter) adk (adenylate kinase), aroE (shikimate dehydrogenase), fumC (fumarate hydratase), gdh (glucose-6-phosphate dehydrogenase), pdhC (pyruvate dehydrogenase subunit) and pgm (phosphoglucomutase). These genes are coding proteins required in the upkeep of the bacteria, and are constantly expressed [18].

Horizontal gene transfer is a common occurrence in the *Neisseria *genus [19], which creates a highly diverse gene pool, and large numbers of genetically heterogeneous strains are constantly created within serogroup B *Neisseria meningitidis *[20], especially at the outer membrane protein level whereby a variety of combinations are present. Epidemics are often due to a select number of hypervirulent clonal complexes [21], which are defined as closely related groups of isolates in which all sequence types (STs) are linked to at least one other single locus variant (SLV) also belonging to the clonal complex. In general, STs can be grouped into three categories: global, related and novel sequence types.

This study concentrates on analysing the distribution and heterogeneity of hypervirulent complex B meningococci causing IMD through the analysis of available epidemiological and MLST data on a localized as well as worldwide scale.

## Methods

**Figure 2:**
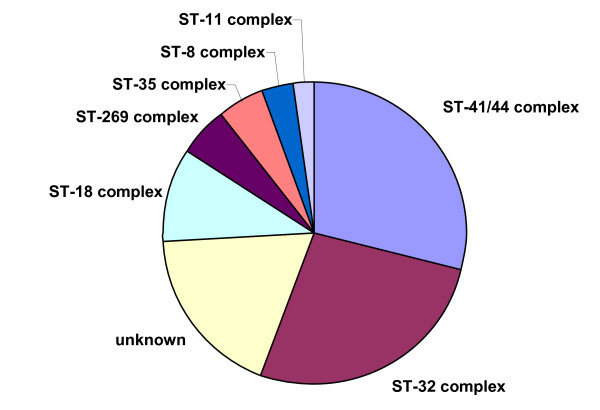
**Distribution of most common ST complexes for serogroup B IMD**. Prevalence of the main serogroup B meningococcal disease clonal complexes found in the MLST database, accessed July 2009.

A systematic review aimed at identifying reports, studies and data concerning serogroup B IMD and the epidemiology attached to it was carried out. International, national and regional websites were consulted for data. Search terms included meningococcal meningitis, *Neisseria meningitidis*, country names, serogroup ACWY and B epidemiology, accessed through Pubmed http://www.ncbi.nlm.nih.gov/pubmed/, as well as government public health and statistics sites for individual countries and regional databases. Exclusion criteria for data was implemented when figures for total case numbers provided were not clear concerning the inclusion of all bacterial meningitis (for example *Streptococcus pneumoniae *and *Haemophilus influenzae *type B) versus meningococcal meningitis (caused by *Neisseria meningitidis*) alone. Data from this review were used for figures 1, 2 and in table 1.

**Table 1:** STs of meningococci which have recently emerged

Country	Japan	Korea	China	Brazil
**Related to widespread clonal complexes**	ST 44 to ST 2055 ST 23 to ST 2038 ST 32 to ST 2145	ST 41/44 to ST 6667	ST 44 to ST 5635 ST 230 to ST 5626 ST 658 to ST 5644	ST 32 to: ST 639 ST 3764 ST 3765 ST 3768 ST 3779 ST 3773 ST 3774 ST 3778 ST 3776 ST 3769 ST 3775 ST 35 to ST 3771 ST 269 to ST 3772

**Novel clonal complexes**	ST 2046 ST 2149 ST 2032		ST 5666 ST 5615 ST 4821	ST 3766 ST 3767 ST 3777 ST 3779 ST 3780 ST 3781 ST 3782

List of novel and related to widespread clonal complexes STs in selected countries

**Figure 3:**
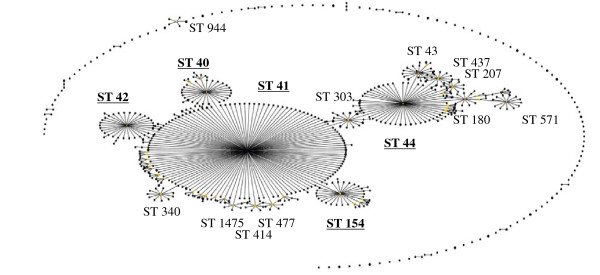
**eBurst representation of IMD clonal complex 41/44**. Each dot represents a ST. Distance from each ST to the founding ST located in the middle of a cluster, indicates diversity level. Dot distance shows difference in single locus variants. Only major STs are labelled.

**Figure 4:**
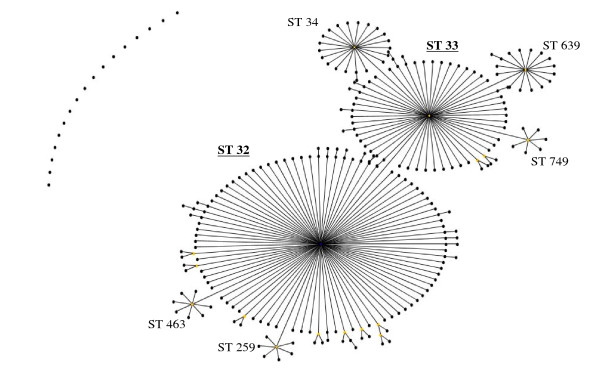
**eBurst representation of IMD clonal complex 32**.

**Figure 5:**
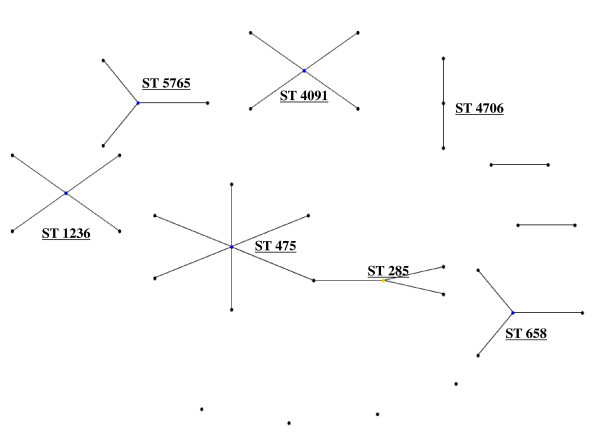
**eBurst representation of IMD clonal complex 11**.

**Figure 6:**
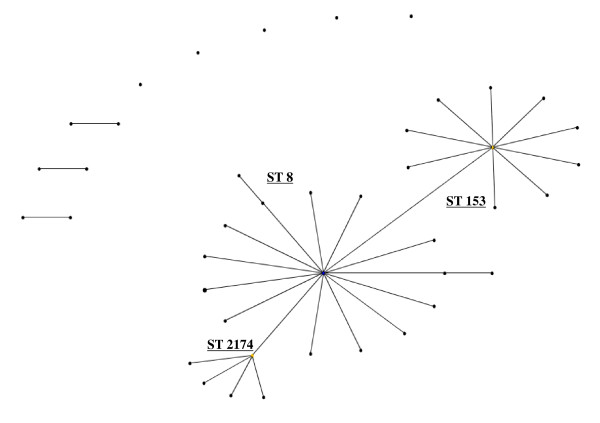
**eBurst representation of IMD clonal complex 8**.

**Figure 7:**
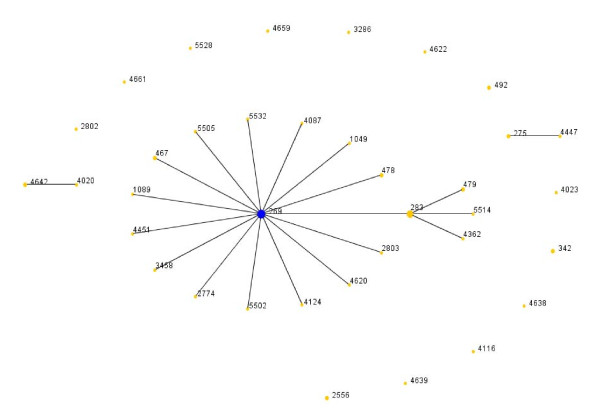
**eBurst representation of IMD clonal complex 269, 1990's**.

**Figure 8:**
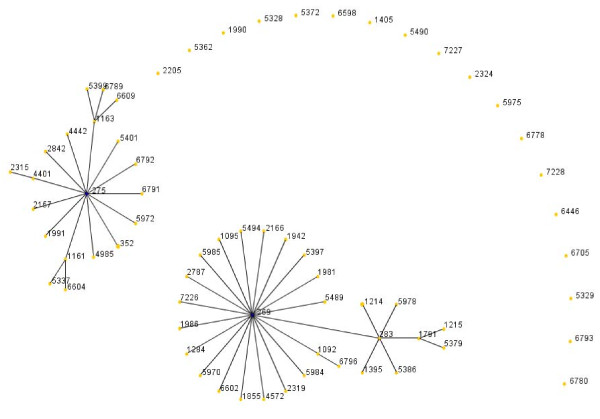
**eBurst representation of IMD clonal complex 269, post 1990's**.

The MLST website [22] was searched for invasive serogroup B data by including the search terms for the desired clonal complexes (ST 41/44, 32, 11, 8 and 269), country (all countries where data was available), species (*Neisseria meningitidis*), serogroup (serogroup B), disease (meningococcal meningitis), epidemiology, age and year, whilst excluding the search terms: carriage, other species and other serogroups (A, C, W135, Y, other and unknown). The data was then sorted down to these specific fields: clonal complexes (STs 41/44, 32, 11, 8 and 269) and entered into the program eBurst V3 http://eburst.mlst.net/[23] in order identify mutually exclusive groups of related genotypes in the population, as well as identifying the founding ST of each group, including the effect of collection years (pre 1990s and post 1999). Data from the MLST analysis were used for figures 3, 4, 5, 6, 7 &8.

The final traditional hypervirulent clonal complex of serogroup B is the ST 8 cc (Figure 6), which is also distributed on a global scale. The STs 8, 153 and 335 are the most common STs in this group.

Further hypervirulent clonal complexes associated with the B capsule have emerged, this include the ST209 cc found in Canada, specifically strain B:17:P1.19 [25]. Interestingly, a comparison of strains belonging to the ST269 cc during the 1990s or after 1999 (Figure 7 &8) in the United Kingdom showed that although the main STs were present in both time frames, the number of different STs was higher among the strains isolated post 1999, as was the percentage of antibiotic resistant strains (penicillin, sulphonamide, ceftriaxone, chloramphenicol, cefotaxime, rifampicin and ciprofloxacin ).

## Results

The most common ST complexes for serogroup B invasive meningococcal disease found when analyzing the distribution and the frequency of the STs recorded in the MLST database, accessed in July 2009, are seen in (Figure 2). Note that not all countries report their information to the MLST database, and although evidence suggests that the prevalence of complexes shown in figure 2 are in correct hierarchy, not all STs are sent and therefore analysed in the MLST database.

Hypervirulent clonal complexes exist for all serogroups responsible for IMD, with the ST 41/44, 32, 11 and 8 also known as lineage III, ET-5, ET 37 and the A4 cluster respectively, being responsible for the majority of IMD in serogroup B [24]. Another emerging hypervirulent clonal complex, ST 269, well documented in Law et al., 2006 [25], is also represented in (Figure 2).

Figures 3, 4, 5, 6, 7 &8 show the founding genotype of each clonal complex and the relationship between the main cluster and the different strains according to the distance shown by the connecting lines. Each dot represents an ST, whereby STs present at one dot distance symbolize a Single Locus Variant (SLV), and the dots located at two distances are the Double Locus Variants (DLV). Where further larger clusters are present, this would indicate that a primary founder has diversified and produced its own SLVs as described in http://eburst.mlst.net/v3/instructions/3.asp.

The ST41/44 complex (Figure 3), with over 1000 documented STs is the most diverse clonal complex associated with serogroup B meningococcal disease. STs 40, 41, 42, 43, 44, 45, 146, 154, 170, 303, 437, 1403 and 3346 are the most widespread STs on a geographical level (data not shown). Importantly, it was responsible for a large epidemic in New Zealand caused by strain type B:4:P1.7-2,4 [21]. Highlighted in (Figure 3), the most common STs in the ST41/44 complex were 41 followed by 44, 42, 40 and 154. This common complex has caused epidemics in The Netherlands and New Zealand, and has also been the dominant cause of IMD in Ireland, Belgium, and Italy [3,26].

The ST 32 complex (Figure 4) has been responsible for several large outbreaks in Iceland, Norway, France Denmark, the Netherlands, United Kingdom [27-30]. It is also spreading to Central and Southern American regions affecting Cuba, Chile and Brazil [7,31,8]. As mentioned above, it is also the ST clonal complex responsible for the hyperendemic situation in the Seine Maritime department in France, caused by the phenotype B:14:P1-7,16 as well as in Oregon, USA where the phenotype B:15:P1.7,16 has caused the majority of IMD in the region. In this clonal complex, the most wide spread strains belong to STs 32, 33, 34, 259, 265, 343, 463 and 749.

The ST 11 clonal complex (Figure 5), although usually associated with serogroup C, and in the Mecca outbreaks with W135 [32-34] also comprises a diversity of STs. In this complex, the number of different ST is comparatively low with the main ST being ST 11.

When comparing the global ST distribution with local datasets such as those found in Taiwan [14], Brazil [35], France [13] and the United Kingdom, similar e-burst structures were observed in general, yet on a local scale, the main difference was seen in the amount of clusters and STs present as well as their diversification patterns. In general there was an increase in number of different STs seen with passing time. As shown in (Figure 7 &8), related or novel STs for serogroup B arise regularly during time, as reported in China [36], Korea [37], Japan [15], New Zealand [21] and Brazil [35] as seen in Table 1.

This well documented process brings to attention the highly genetically diverse nature of serogroup B strains [20]. Several hypotheses have arisen including the effects of carriage processes [21], vaccine replacement [25] or the presence of new allelic recombinations [38]. In this light, vaccine development efforts need to be tailored to this phenomenon.

## Discussion

As a bacterium of a highly recombining nature, allowing for a vast genetic variability [39], and the emergence of hypervirulent strains, challenges have remained persistent in the development of effective prevention and control methods for this disease. As seen in Table 1, there is a continued output of related and novel STs occurring worldwide. There are several main hypervirulent strains as seen in (Figure 2), due to the commensal nature of *N. meningitidis*, and as seen in figure 3, 4, 5, 6, 7 &8 and documented in many studies, the development of new strains is likely to continue. Perhaps by studying the patterns of ST distribution, the identification and targeting of several STs for vaccine purposes could be achieved, and highlight the fact that local vaccines would not be a long term solution.

Still a poorly understood aspect of meningococcal disease is the role of carriage versus invasive disease. As described in [40], even within clonal complexes, differences in carriage versus invasive disease causing groups exist. For example as seen in cases involving the Czech Republic, Greece, and Norway, three main STs in serogroup B have been associated with invasive disease: ST32, ST 269 and ST18, whilst ST 35 was distinctively more linked to carriage isolates[41]. Additionally, it has been shown that carriage isolates seem to be more diverse than invasive ones [36].

Even if an effective vaccine was developed targeting the major antigens in serogroup B invasive disease causing STs, there have been reports of capsule replacement as seen in Italy [42] from serogroup C to serogroup B (ST11). Capsule switching has also been reported in two distinct scenarios, firstly during an epidemic as seen in the Czech Republic [43] with the majority of replacement being from C:2a:P1.2,5. to B:2a:P1.2(P1.5), or after an immunization campaign as described in Canada whereby serogroup B ST-269, B:17:P1.19 emerged from C:2a:P1.5,2 after a vaccine against serogroup C disease was distributed [25].

## Conclusions

Any potential vaccine against serogroup B disease would need to target areas of the agent which are not linked by serogroup alone but more specifically to characteristics affecting the bacterium itself as targeted by a general protein based vaccine. Additionally, the pressure of serogroup B dominance seen in many countries at present combined with the presence of antibiotic resistance, vaccine development needs to target areas of the bacterium which tackle this widespread and heterogeneous aspect of meningococcal meningitis disease.

## Competing interests

JL is affiliated to Novartis Vaccines and Diagnostics which is a producer of vaccines. The authors declare that they have no competing interests.

## Authors' contributions

VR conducted the research and analysis, LJS helped draft the manuscript. All authors read and approved the final manuscript.

## Pre-publication history

The pre-publication history for this paper can be accessed here:

http://www.biomedcentral.com/1471-2334/10/175/prepub
